# Supplementary material for: Structural dynamics and neural representation of wing deformation
Source: Proc Natl Acad Sci U S A. 2025 Nov 13;122(46):e2518032122. doi: 10.1073/pnas.2518032122 (PMC12646313; doi:10.1073/pnas.2518032122)
Supplement: Supplementary file 1 — Appendix 01 (PDF) [file pnas.2518032122.sapp.pdf]

Supporting Information for

Structural dynamics and neural representation of wing deformation.

Alexandra M. Yarger, Masateru Maeda, Igor Siwanowicz, Haruhiro Kajiyama, Simon M. Walker, Richard J. Bomphrey, & Huai-Ti Lin

Alexandra M. Yarger & Huai-Ti Lin
Email: a.yarger@imperial.ac.uk, h.lin@imperial.ac.uk

This PDF file includes:

- Supporting text
- Figures S1 to S9
- Table S1
- Legends for Movies S1 to S5
- SI References

Other supporting materials for this manuscript include the following:

- Movies S1 to S5

Supporting Information Text

Model choice

To demonstrate how morphological computation contributes to the tuning of biological sensory systems, dragonflies are an ideal model. Their large, highly deformable wings are easy for structural measurements and their wing afferent signals are accessible for electrophysiological recordings. Furthermore, while all flying animals have deformable mechanosensitive wings, dragonflies are among the most agile fliers and their wings are frequently used as models for aerodynamics and wing mechanics(37, 76–84). The dragonfly wings' mechanics naturally limit their range of deformation, but there is still a large available state-space significant for flight control.

Tegula complex

In locust(1, 2) and hawkmoth(3) a mechanosensory structure on the hindwing - called the tegula - influences forewing motor control. The tegula plays an important role in the generation of rhythmic flight motor patterns in both insects but has not previously been described in dragonflies. We identified a tegula-like structure called the 'tegula complex' which comprises a hair plate and CS field (Fig. S8D). The tegula complex, like the tegula, may be responsible for maintaining wing synchrony in dragonflies. Forewing nerve ablated dragonflies are still able to maintain synchrony between their fore- and hindwings, albeit at a lower frequency, indicating that the hindwings (and hindwing tegula complex) may be responsible for maintaining wing synchrony. Afferents from the tegula complex were not recorded here but may be of interest for future studies investigating mechanisms for wing synchronization.

Phase shifts

Phase time shifts that result from changes in displacement amplitude (Fig. 3C) are descriptive of a single feature only and those features have been extracted from the overall displacement pattern. Larger amplitude bending does not scale equally with twist or camber, and here the displacement speed has also been normalized across amplitudes. This may partially explain why we often see a phase advance rather than a phase delay with increased amplitudes. Alternatively, large amplitude bending is typically caused by large aerodynamic loading. Such passive deformation can be characterized by a wave propagating tip-to-base, in contrast to the deformation driven by the flapping musculature with a base-to-tip propagation. Depending on the location of the exact strain sensor being recorded, the phase timing will shift according to the contribution from these two waves. Regardless of the specific physical mechanism, it is important to consider that the overall pattern of motion is not identical for every amplitude. However, because the stimulus space is constrained by the wing structure, the possible deformations are still limited to a finite range of patterns that can be described by bend, twist, and camber. Any motion outside of the natural wing state will necessarily activate additional sensors that can encode the deformations not represented within the natural range.

Perturbation-activated sensors

In general, individual variation and adaptability exist because the nervous systems is able to produce appropriate outputs using a variety of different mechanisms(4, 5). We did not characterize the selectivity of perturbation-activated sensors as it is outside the scope of the current study. It requires extensive cataloguing of responses across conditions, morphologies, and individuals. Selectivity and encoding strategies are unlikely to be identical between individuals(5) and input sensitivity is also likely to depend on context e.g. structural changes or behaviour state(6–8). The large number of active units also prevents accurate sorting with the current recording preparation. Future experiments will aim to resolve more units simultaneously

for characterizing these sensors' selectivity and other factors including sensor redundancy and adaptability.

Deformation Frequency components

Airflow induces consistent wing oscillations as a result of the structural and material properties of the wing. The natural frequency of the wing (Fourier transform dominant frequency of mean displacement of entire wing) across animals was 135+/-23Hz. Faster air speeds resulted in slightly higher frequencies (18.6+/-15.2Hz increase from 1-3m/s, N=5 animals). The dominant frequencies of the PCs were the same as the mean displacement frequency (Fig. S6). Vibration induced deformations have higher frequencies of twist and camber motion.

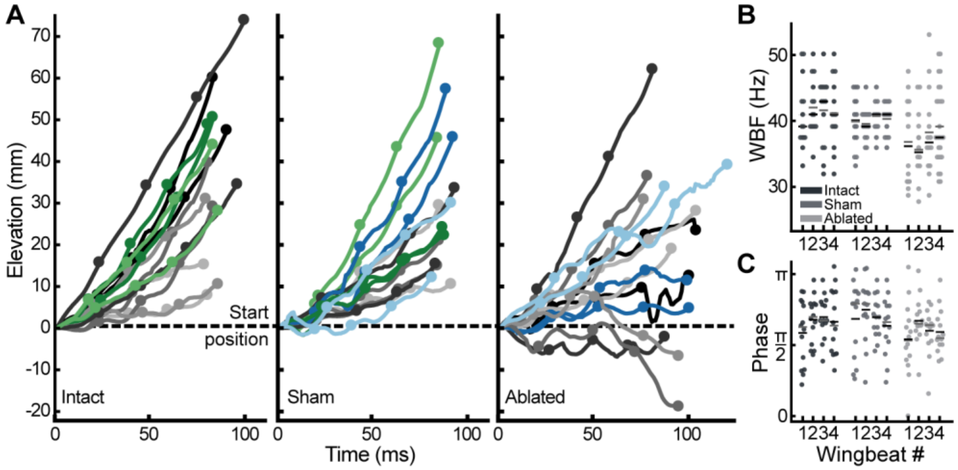

Fig. S1. Nerve ablation. A) Take-off elevation over the first four wingbeats. Different shades represent different animals. Circles mark start of forewing downstroke. N=7 animals, 2 trials per condition. Two animals appear in both intact and sham (green) and two in both sham and ablated (blue). B) No significant differences in WBF between wingbeats within each condition. Grey and black bars show mean and median respectively. Ablated WBF significantly lower than intact (P=0.00002) and sham (P=0.0024). No significant difference between intact and sham WBF. C) No significant differences in the hindwing phase relative to the forewing between wingbeats within each condition. No significant differences between all conditions. Data resolution results from video frame rate (1000 fps).

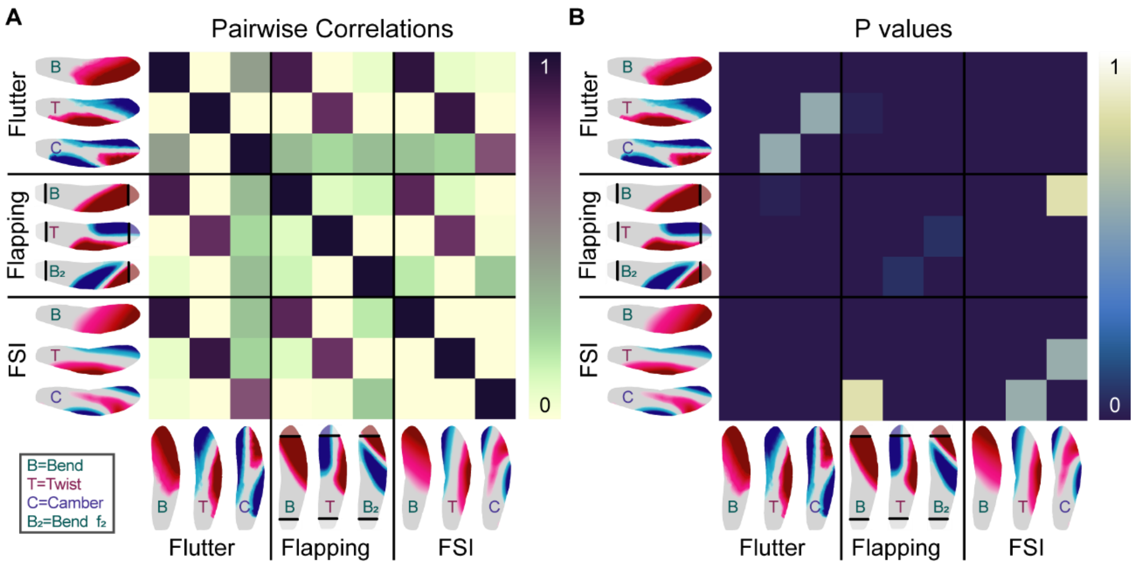

Fig. S2. Pairwise correlations of displacement maps. A) The bend pattern is highly correlated across all conditions. Twist is also highly correlated between conditions. Camber is only correlated between Flutter and FSI because camber could not be measured during flapping. B) All highly correlated pairs were significant. The only non-significant correlations were between combinations with low correlation values (e.g. flapping bend & FSI camber).

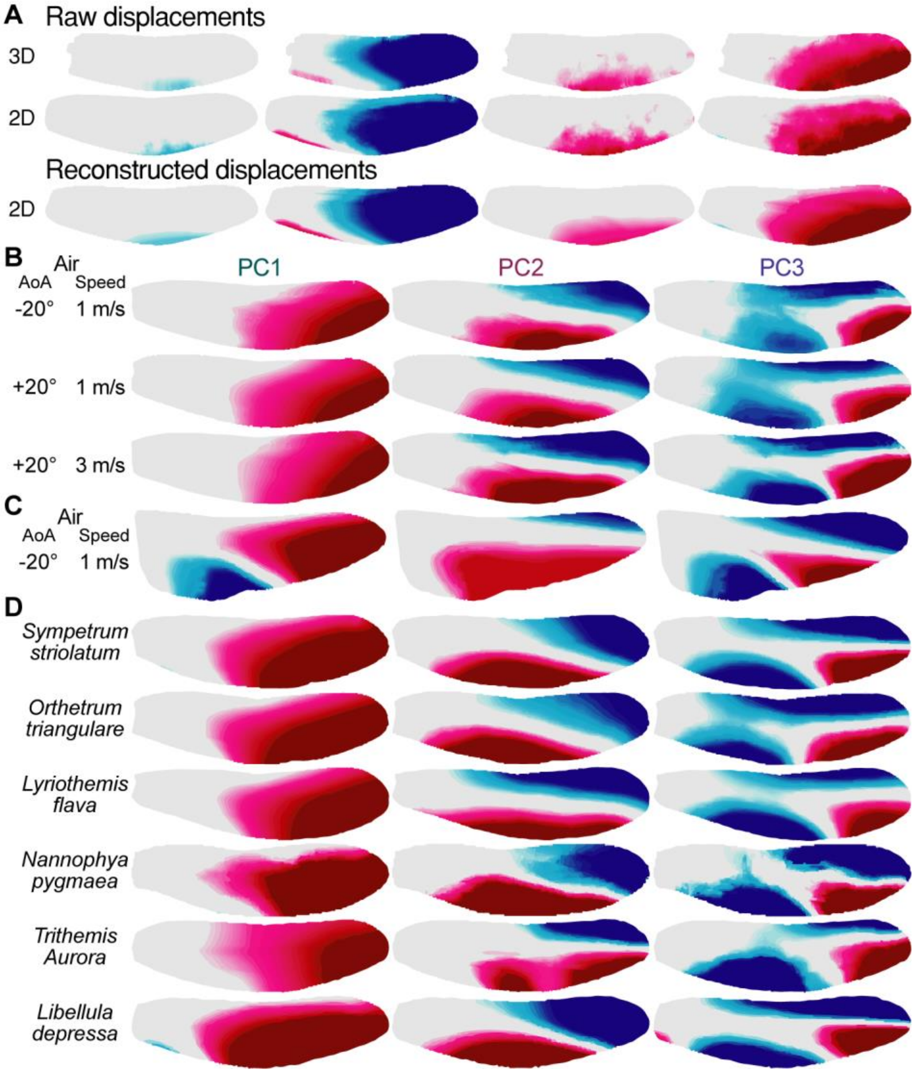

Fig S3. Wing displacement PCs. A) Example frames of two camera 3D measurements of displacement magnitude (top), single camera dorso-ventral displacement measurements (middle) and displacement reconstructed from PCs 1-3 (bottom). B) Angle of attack (AoA) and air speed do not influence PCs. Example PCs calculated from 7000 frames. C) Hindwing PCs are similar to forewing PCs. PCs calculated from 5000 frames. D) PCs are the same across dragonfly species tested. PCs calculated from 100-1000 frames. All wings filmed at 1000 fps and resized to equal wingspan and cord width for easier comparison.

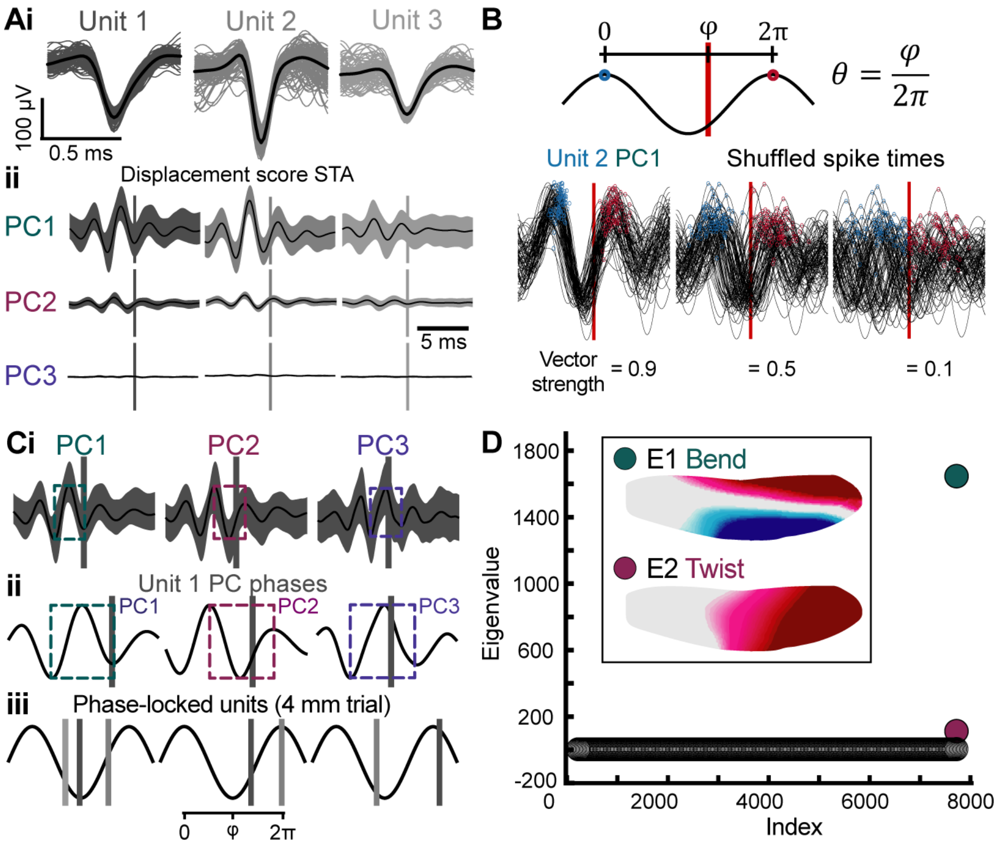

Fig. S4. Phase measurements. A) Sorted units shown in Figure 3 (i) and their associated PC STAs (ii). B) Phase (q) is calculated by measuring the relative time (j) between oscillation peaks normalized to radians. Phase-locked units are identified by calculating vector strength (see Eq.1). Units with vector strength above 0.5 are classified as phase-locked. C) Unit 1 phase (vertical bars) relative to PC score STAs (i) normalised to radians (ii). All phase-locked units from airflow induced deformations with 4 mm mean displacement (iii; see also Fig. 3). D) Significant features (E1-E2) identified through covariance analysis of the spike-triggered raw displacements. Camber was notably not present, indicating that this mode is less likely to be represented in the neural signal. This result was consistent across units tested.

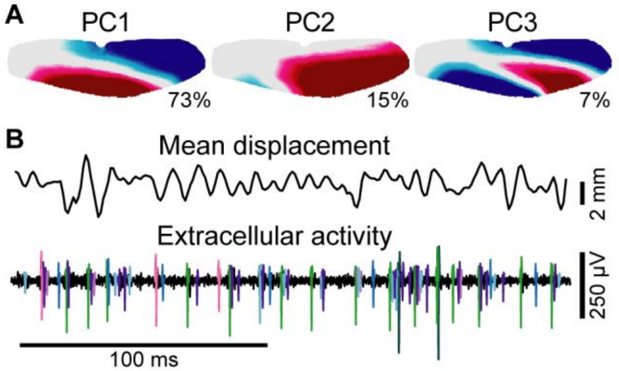

Fig. S5. Airflow playback vibration. A) Airflow playback vibration induced deformation PCs. B) Airflow playback result in moderately increased extracellular activity compared to airflow induced displacements. Colours indicate different units.

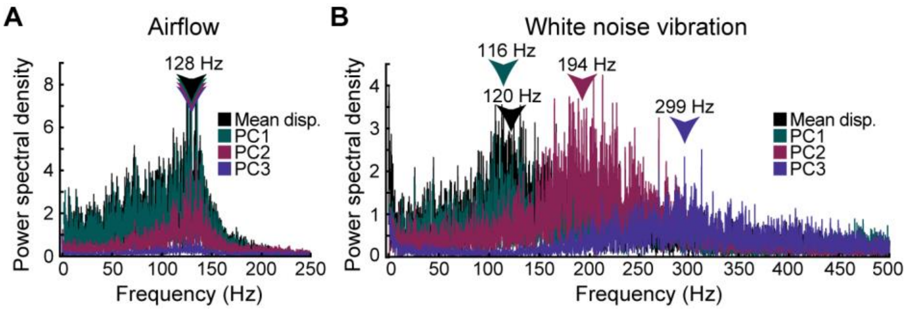

Fig. S6. Displacement frequency components. Frequency components of mean absolute displacement of the entire wing and PC scores (PC1-bend, PC2-twist, PC3-camber) for airflow (A) and white noise vibration (B) stimuli.

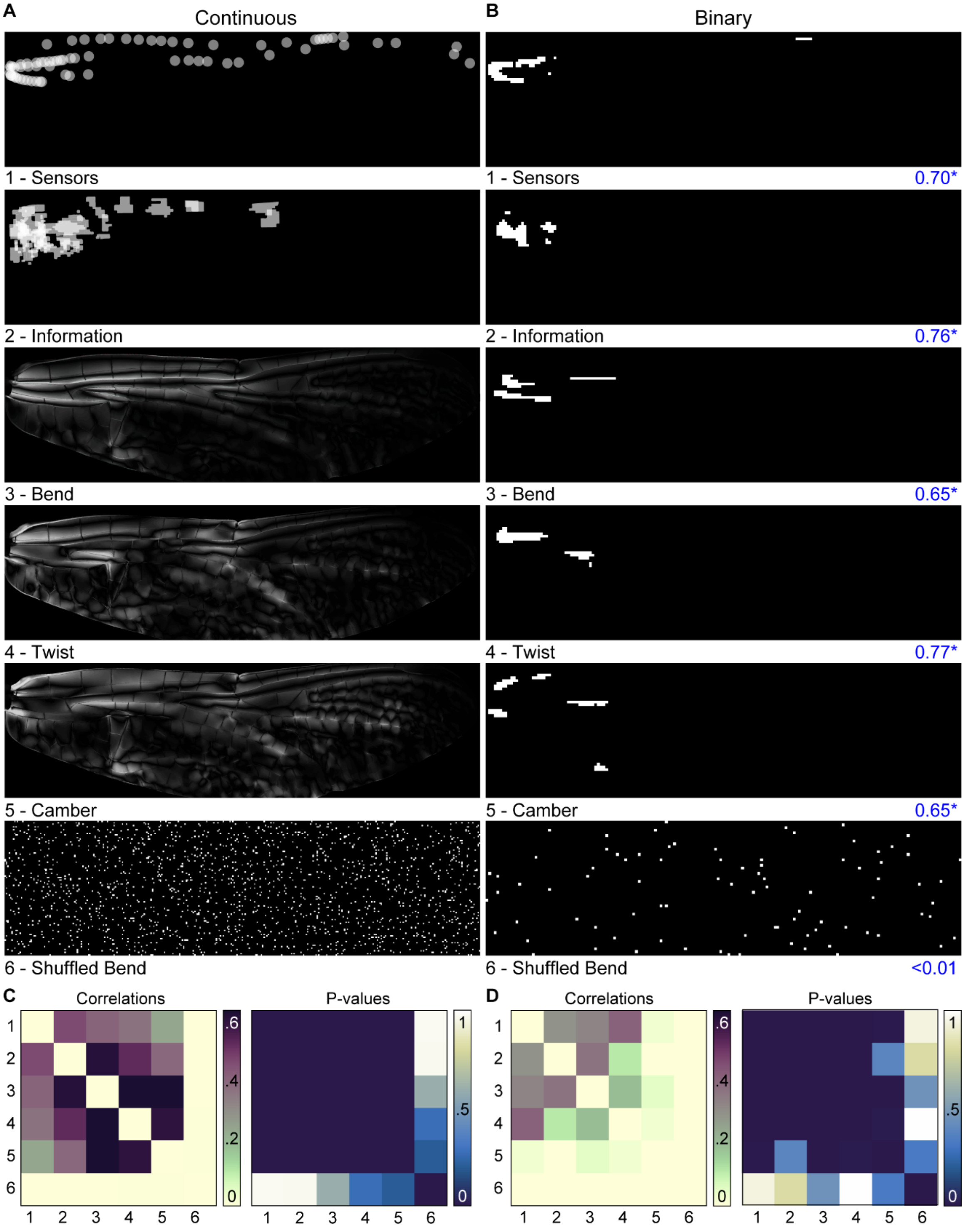

Fig. S7. Hotspot analysis. A) Sensor, information, strain maps, and shuffled bend stain map converted to continuous greyscale images. B) Hotspots from A defined by 99th percentile plotted as binary images. Spatial autocorrelation (Moran's I) values for each image shown in blue. Values >0.5 suggest strong clustering. Significant values indicated by *. C) Correlation statistics for continuous images (A). D) Correlation statistics for binary images (B).

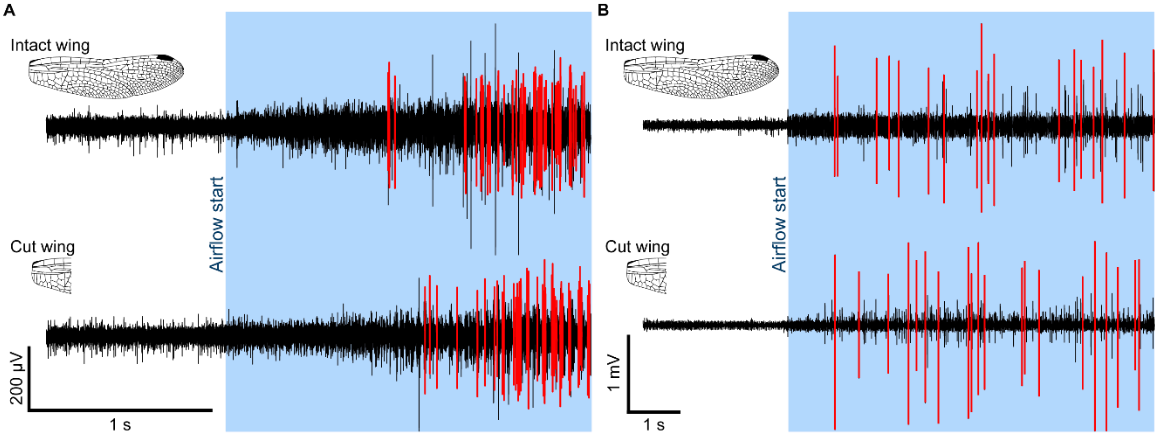

Fig. S8. Sensor activity remains after distal portion of wing is removed. Extracellular recording from *Orthetrum cancellatum* (A) and *Celithemis elisa* (B) wing nerve in response to airflow with wing intact (top) and cut (bottom). Large sensory units highlighted in red.

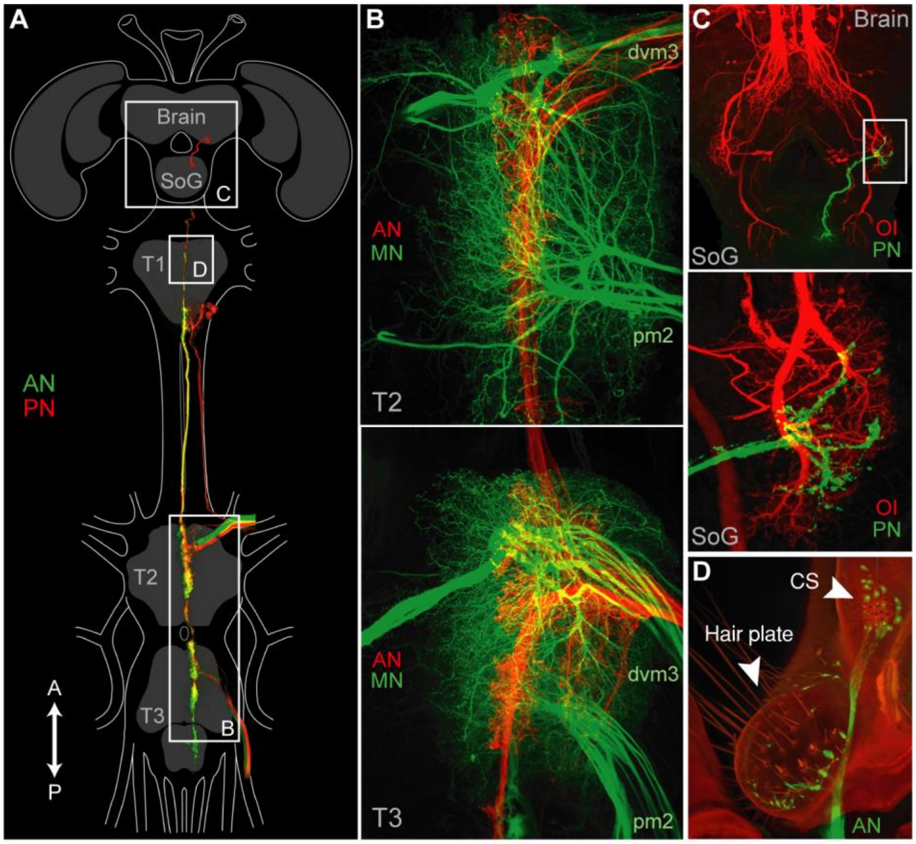

Fig. S9. Wing mechanosensor neuroanatomy. A) Anterior (AN) and posterior (PN) wing nerves project ipsilaterally within T2 & T3. PN projects to T1 and brain (panel C) and a subset of AN afferents from the tegula complex (panel D) project to T1. B) AN projects to wing motor neurons (MN) dvm3 (pronation) and pm2 (anti-overpronation). C) PN projections to secondary ocellar interneurons (OI) in the posterior deutocerebrum. D) Hair plate and CS comprise the tegula complex.

Table S1.

Comparison	Between wingbeats			Between conditions		
	Intact	Sham	Ablate	Intact vs. sham	Sham vs. ablate	Intact vs. ablate
Phase	0.58	0.47	0.75	0.058	0.023	0.57
WBF	0.94	0.63	0.49	0.016	0.0024*	0.000017**

Multiple comparisons P values for nerve ablation experiments.

Movie S1.

High-speed video of a dragonfly taking off in its natural environment followed by the same behaviour captured in a nine-camera, free-flight arena. Wing kinematics of freely flying dragonflies were tracked (left) and reconstructed in 3D (right). Wing displacements (blue-upward, red-downward) were measured throughout the stroke cycle during free-flight and simulated gliding (fixed-wing airflow-induced displacements). Wing displacements were measured while simultaneously recording the neural activity of wing mechanosensory afferents (strain sensors). 99% of the measured displacements are comprised of three principal components (bend, twist, and camber) and different sensors (coloured spikes) are activated by different ratios of those components. Micro-CT scans of the wing were used to construct a high-fidelity morphological model of the dragonfly forewing. Fluid-structure simulations of gliding flight show detailed measurements of wing displacement and strain. The regions of the wing that experience the largest strains overlap with the identified locations of the strain sensors (that encode the principal deformation components).

Movie S2.

Free-flight kinematics: Example flight trajectory includes three wingbeats where a roll manoeuvre is performed during the last wingbeat.

Movie S3.

Example intact vs. nerve ablated take-off. Wingbeat frequency decreases after nerve ablation and animals often abandon take-off attempts once airborne.

Movie S4.

Free-flight deformations. Displacement field maps generated from tracked leading and trailing wing edges.

Movie S5.

Global displacement of FEA modes. Modes 1 & 2 show bend and twist. Modes 3-4 show camber.

SI References

1. E. Gettrup, Sensory Regulation of Wing Twisting in Locusts. *Journal of Experimental Biology* **44**, 1–16 (1966).

2. H. Wolf, The Locust Tegula: Significance for Flight Rhythm Generation, Wing Movement Control and Aerodynamic Force Production. *Journal of Experimental Biology* **182**, 229–253 (1993).

3. M. A. Frye, Effects of stretch receptor ablation on the optomotor control of lift in the hawkmoth Manduca sexta. *J Exp Biol* **204**, 3683–3691 (2001).

4. E. Marder, A. L. Taylor, Multiple models to capture the variability in biological neurons and networks. *Nat Neurosci* **14**, 133–138 (2011).

5. J. A. Supple, *et al.*, Binocular Encoding in the Damselfly Pre-motor Target Tracking System. (2020). https://doi.org/10.17863/CAM.48011.

6. K. D. Longden, T. Muzzu, D. J. Cook, S. R. Schultz, H. G. Krapp, Nutritional State Modulates the Neural Processing of Visual Motion. *Current Biology* **24**, 890–895 (2014).

7. S. J. Huston, H. G. Krapp, Nonlinear Integration of Visual and Haltere Inputs in Fly Neck Motor Neurons. *J. Neurosci.* **29**, 13097–13105 (2009).

8. D. Rien, R. Kern, R. Kutz, Octopaminergic modulation of a fly visual motion-sensitive neuron during stimulation with naturalistic optic flow. *Frontiers in behavioral neuroscience* **7** (2013).
